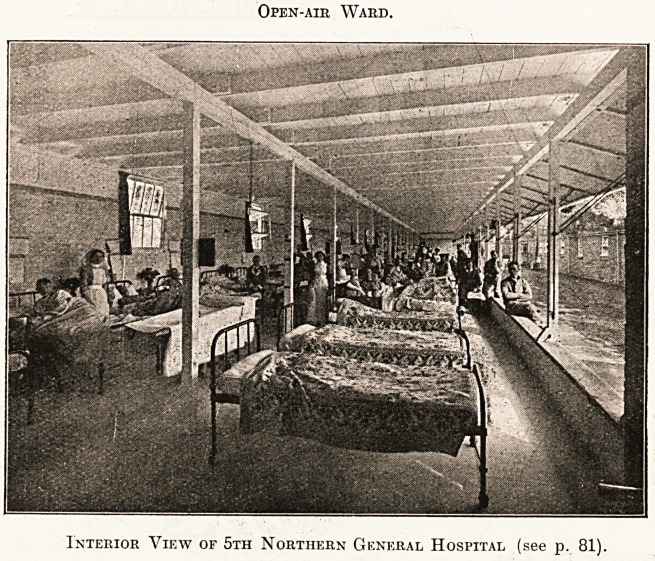# The Third London General Hospital, Wandsworth Common, S.W.

**Published:** 1915-10-23

**Authors:** 


### THE THIRD LONDON GENERAL HOSPITAL,
### Wandsworth Common, S.W.

This hospital is one of the four large General Hospitals administered in London by the Territorial Force Associations of the County and City of London, and was originally intended to be a 500-bed unit housed in the permanent buildings of the Royal Patriotic Fund Corporation at Wandsworth, who very generously handed over the whole of its institution for female orphans of soldiers and sailors on the outbreak of the war to the Territorial Association. The hospital has since grown to a 1,500-bed unit, and will probably be still further extended in the near future. It was originally intended to house the unit in the Patriotic School buildings, but it was found to be impracticable to get more than 250 beds, and hut used as children's infirmary. There are also the O.C.'s private residence, and new rooms for resident officers. In twelve wards (originally the children's dormitories) accommodation is found for about 250 beds. All the buildings have been modified to suit them to hospital requirements, the work including up-to-date sanitary appliances and baths, with alterations to the whole of the windows throughout so as to provide increased ventilation.

### The Temporary Huts (1,300 Beds).

The accommodation in the temporary huts is as follows: Thirty-six wards for about 1,300 beds; two resident officers' huts, with cubicles and sanitary appliances accommodation was put up for an additional 300 beds on the field to the north of the schools connected to same by a corridor; afterwards the portion of Wandsworth Common adjoining the schools was taken over, and the two further additions built thereon.

The hospital is illustrated by a block plan and two types of ward huts, the latter being referred to later.

### The Hospital Proper.

The hospital as at present existing includes the following accommodation: In the original permanent building (shown on block plan by hatched lines) are placed the O.C., the registrar, with clerk and general administrative offices, nurses' dining-hall, and kitchen. There is also a recreation hall for patients, laundry, dispensary store, engineers' shops, high-pressure disinfecting plant and steam sterilisers, laboratory, *post-mortem* room and mortuary. The officers' hospital is in a building originally for 180 officers; a large officers' and men's pack store; the steward's store; linen store; dirty linen store; a central bath-house with boiler-house attached and nineteen bath cubicles; a large kitchen (36 ft. by 100 ft.), fitted with gas cooking apparatus throughout; a service counter, scullery, and larders; the dining-hall, to seat 500; the retiring rooms for matrons, nurses, and doctors, and sanitary accommodation to same. There are two large operating theatres capable of accommodating four tables, and sterilising-rooms, etc.; two *x*-ray rooms, dark room, and electric treatment room; water tower, with 4,000 gallons water storage; ten orderly huts, accommodating 220 orderlies, with dining-hut, kitchen, etc., all in a barrack yard; large incinerator by Manlove and Alliott. There are corridors 8 ft. wide throughout connecting the various hospital buildings.

As before mentioned, we illustrate a block plan and two types of ward huts, one being for officers and the other for privates. The floor and cubic space per bed is sufficient, although naturally not what one is accustomed to find in a ward devoted to serious surgical cases.

THE OFFICERS' WARDS.

The figures for officers' wards are as follows: Wards, 85 ft. by 22 ft.; height, 11 ft. (partly ceiled in roof); floor space per bed, 90 ft. super.; cubic space per bed, 1,125 ft. In these wards the beds are properly spaced between each pair of windows, the end beds having ensured to them proper lighting and ventilation by small windows at the ends of the wards. The windows are of the hopper type, each being in two heights and the sashes falling inwards. The wards are heated with stoves placed in the centre, the flue pipe in each case passing through the roof. This flue is encircled by a ventilating flue. This arrangement, taken in connection with the fact that part of the roof space proper is in the ward, should serve to promote good ventilation.

A single bed ward, 18 ft. by 10 ft. 6 in., is provided for special cases near the entrance to the ward block; a cupboard for linen, a scullery and nurses' duty room, fitted with dresser, cupboard, sink, gas boiler, and gas stove. When, however, to these fittings is added a table, this office must be somewhat crowded, and we think it a pity that it could not have been increased in size, seeing that it has to do duty for twenty - one patients. A food cupboard is provided opening out of the scullery.

OPEN-AIR WARD.

INTERIOR VIEW OF 5TH NORTHERN GENERAL HOSPITAL (see p. 81).

WARDS FOR WOUNDED SOLDIERS.

The wards for private soldiers marked B B B on plan present some special points for criticism. Although we fully appreciate the value of abundant lighting in any ward, especially one devoted to the purpose of sick and wounded patients, we cannot bestow unqualified approval on the method of forming practically long sides of a ward with glass. Glass is too good a conductor of heat and cold to form an ideal wall for any dwelling for sick or well, and unless some special steps are taken to counteract the downrush of the chilled air on to the heads of the patients on winter nights, we fear it must be most uncomfortable for them. For the rest the arrangements are similar to the wards for officers, except that the special case ward opens into the main ward as well as into the short corridor. Also the bath-room and lavatory, we are glad to notice, are not in a projecting annexe.

THE KITCHENS, STORAGE, AND UNIT WARD.

The storage for linen appears ample. A kitchen is provided with fittings as before described, having a larder and cupboard for brooms opening therefrom.

The two projecting annexes are similarly arranged as to the officers' block, excepting that one is entirely occupied by the sink-room and the other by two w.c.s and a lavatory. The space between the two sanitary annexes is occupied by a verandah, roofed over, and enterable from the end of the ward by means of folding doors. Two beds are placed in this verandah. An elevation of the end of the ward, showing the verandah, is given. These wards are 100 ft. by 20 ft., each accommodating twenty-eight patients, and the space in the roof is part of the cubic space, as the soffits of the roof timbers are plastered. It may here be mentioned that almost every ward has a verandah provided, on which bad septic cases are treated, and these verandahs are found of the greatest possible value, the men sleeping out in them in all but the worst weather.

THE DETAILS OF CONSTRUCTION.

The buildings generally are constructed with 4 in. by 4 in. timbers, posts being imbedded in concrete bases, 4 in. by 4 in. sills and heads, and 4 in. by 2 in. studs, and the external coverings are with 22-gauge galvanised iron, that to roofs on $\frac{3}{4}$-in. boarding. The three wards occupied by officers are externally covered with metal laths and finished with cement rough cast, the roofs being covered with asbestos cement slating. The internal walls are lined with $\frac{1}{2}$-in. plaster slabs and rendered in Keen's cement, and asbestos sheet linings to roofs. The floors are $1\frac{1}{4}$ in. grooved and tongued boards on 5 in. by 3 in. joists. The walls and soffits of roofs are distempered, and all woodwork is coated with Solignum outside and stained and wax polished inside, including the floors. Every ward has a gas boiler and storage cylinder for hot water, with a gas cooking-stove.

The arrangement of the various blocks on the site is not completely satisfactory, and we could wish that more of the wards had been provided on both sides with their full proportion of morning and afternoon sunlight. We feel it is fair to add that the scheme, not having been worked out as a whole from the commencement, has created many difficulties, the most serious results being traceable to piecemeal planning.

The hospital covers an area of about forty acres, and the cost per bed comes to about £48.

The architect is Mr. J. Pain Clark, A.R.I.B.A., 12 South Square, Gray's Inn, E.C., and it may be mentioned that the scheme has been worked out to a great extent in consultation with Colonel Russell, the chief engineer, London district, and Lieutenant-Colonel Bruce Porter, the officer commanding the hospital.

## Figures and Tables

**Figure f1:**
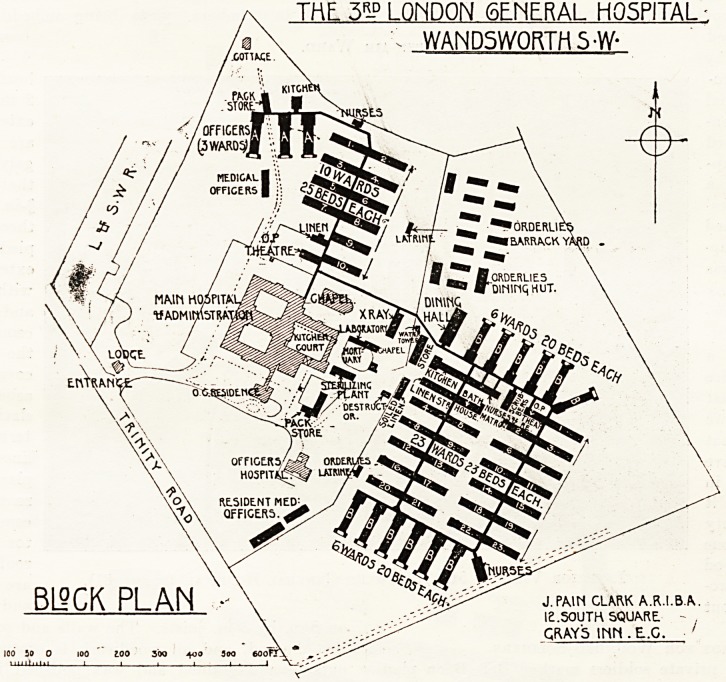


**Figure f2:**